# Older Adult Behavioral Health Issues in New England: Findings from the Healthy Aging Data Reports

**DOI:** 10.1093/geroni/igab046.2872

**Published:** 2021-12-17

**Authors:** Taylor Jansen, Chae Man Lee, Shu Xu, Maki Karakida, Frank Porell, Nina Silverstein, Beth Dugan

**Affiliations:** 1 UMass Boston, Boston, Massachusetts, United States; 2 University of Massachusetts Boston, University of Massachusetts Boston, Massachusetts, United States; 3 University of Massachusetts Boston, Boston, Massachusetts, United States

## Abstract

Behavioral health issues in older adults are prevalent and have negative consequences on quality of life and overall health, yet are often untreated. The present study compares state and local community rates of behavioral health indicators of older adults age 60+ in Massachusetts (MA), New Hampshire (NH), Rhode Island (RI), and Connecticut (CT). For this study rates were calculated from the following data sources: Medicare Current Beneficiary Summary File 2014-2018 (2014-2015 MA, NH, and 2016-2017 RI, CT) and the Behavioral Risk Factor Surveillance System (2013-2015 MA, 2014-2016 NH, 2015-2017 RI, CT). Small area estimation techniques were used to calculate age-sex adjusted community rates for more than 170 health indicators (https://healthyagingdatareports.org/). This research examines disparities in rates across the 4 states for 4 behavioral health indicators: substance use disorder (SUD), tobacco use disorder (TUD), opioid use disorder (OUD), and excessive drinking. Results varied across states with RI reporting the highest rates of substance (7.0%) and tobacco use (10.8%) disorders, CT had the highest rate of opioid use disorder (2.2%), and MA and RI reporting the highest rates of excessive drinking (9.3%). Overall, MA had the greatest disparities in rates for all indicators (SUD: 6.6% (5.35-15.99%); TUD: 10.2% (2.67-24.20%); excessive drinking: 9.3% (5.63-19.98%)), indicating behavioral health disparities by community are most pronounced in MA. This study found behavioral health issues are prevalent among New England older adults and should no longer be overlooked. Furthermore, visualizing the community rates makes disparities evident and may guide resources and services to areas of highest need.

